# Repeated Impact Performance of Carbon Spread-Tow Woven Stitched Composite with Anti-Sandwich Structure

**DOI:** 10.3390/polym17192670

**Published:** 2025-10-02

**Authors:** Minrui Jia, Jingna Su, Ao Liu, Teng Fan, Liwei Wu, Kunpeng Luo, Qian Jiang, Zhenkai Wan

**Affiliations:** 1 Engineering Teaching Practice Training Center, Tiangong University, Tianjin 300387, China; jiaminrui@tiangong.edu.cn; 2 Tianjin and Ministry of Education Key Laboratory for Advanced Textile Composite Materials, Tiangong University, Tianjin 300387, China; sjingna@163.com (J.S.); liuao14745155899@outlook.com (A.L.); fanteng2024@163.com (T.F.); wuliwei@tiangong.edu.cn (L.W.); 3 School of Textile Science and Engineering, Tiangong University, Tianjin 300387, China; 4 KaiBen Carbon Neutrality (Tianjin) New Materials Co., Ltd., Tianjin 301509, China; luo002@yeah.net

**Keywords:** spread-tow woven, stitched composite, impact, structure-property relation

## Abstract

Spread-tow woven fabrics (STWs) have attracted considerable attention owing to their thin-layered characteristics, high fiber strength utilization rate and superior designability, finding wide application in the aerospace field. To meet the application requirements for materials with high specific strength/specific modulus in the aerospace field, this study designed an anti-sandwich structured composite with high specific load-bearing capacity. Herein, the core layer was a load-bearing structure composed of STW, while the surface layers were hybrid lightweight structures made of STW and nonwoven (NW) felt. Repeated impact test results showed that increasing the thickness ratio of the core layer enhanced the impact resistant stiffness of the overall structure, whereas increasing the proportion of NW felt in the surface layers improved the energy absorption of the composites but reduced their load-bearing stiffness and strength. The composite exhibited superior repeated impact resistance, achieving a peak impact load of 17.43 kN when the thickness ratio of the core layer to the surface layers was 2:1 and the hybrid ratio of the surface layers was 3:1. No penetration occurred after 20 repeated impacts at the 50 J or 3 repeated impacts at 100 J. Meanwhile, both the maximum displacement and impact duration increased, whereas the bending stiffness declined as the number of impacts increased. The failure mode was mainly characterized by progressive interfacial cracking in the surface layers and fracture in the core layer.

## 1. Introduction

Spread-tow woven fabrics (STWs) exhibit fewer interlacing points and reduced yarn crimp. This characteristic enhances resin penetration efficiency and prevents composite defects such as resin-rich zones formed at warp and weft intersections [1,2]. STWs offer enhanced flexibility in tailoring composite performance, owing to their reduced thickness and excellent drapability. Therefore, carbon STW fabrics exhibit significant potential for widespread use in engineering fields such as aerospace, automotive, and marine applications [3].

As a severe natural hazard, hail presents a major challenge to the operational safety and service life of aviation materials, often leading to premature failure in engineering practice [4]. The short-duration, high-repetition nature of hail impact makes the interlaminar toughness of laminated composites a key focus for damage tolerance under repeated impact conditions [5,6,7]. Repeated impact testing is conducted using a light air cannon or drop-weight impact device to apply multiple consecutive impacts at specified energy or velocity levels, thus simulating the short-duration and high-frequency loading characteristics of hail impact [8]. The test typically assumes an extreme scenario, concentrating all impacts at a single point to enable the observation of progressive damage evolution. This method has been extensively used in studies of laminated composites [9,10]. Research has demonstrated that while the stacking sequence significantly affects resistance to repeated impacts, quasi-isotropic layups generally provide better impact resistance [11]. To some extent, STW fabrics provide superior impact damage tolerance compared to traditional 2D woven fabrics in laminated composite. However, the increased overall density resulting from STW not only reduces the specific strength and modulus of the composites but also leads to over-engineering and material waste in non-load-bearing regions. To meet the growing demand for lightweight, high-strength materials and achieve goals of cost reduction and efficiency improvement in engineering, sandwich composites with lightweight core structures have been widely adopted. STWs enable more lay-up designs of different structures, such as spiral plies [12], overlapping plies [13], and hybrid lay-ups [14,15,16], which expand the design possibilities for composite structures.

The sandwich structure is a typical lightweight yet high-strength configuration characterized by its use of load-bearing face sheets and a lightweight core, which together provide high bending resistance at a low overall weight. However, the placement of the load-bearing layers far from the geometric center makes the sandwich structure susceptible to failure modes such as delamination and debonding during repeated impacts, consequently compromising its structural performance [6,17,18]. To address this, an anti-sandwich structure [19] was designed, featuring a high-stiffness load-bearing core and utilizing low-stiffness, lightweight layers on the outer surfaces. Compared to traditional STW lay-up, anti-sandwich structures not only retain high strength and stiffness but also effectively reduce the overall structural density through graded density distribution across the layers, thereby significantly improving the specific strength and specific stiffness. The differences in density and stiffness within the anti-sandwich structure are mainly achieved by introducing nonwoven (NW) layers. The low density and fiber-bridging mechanism of carbon fiber NW layers effectively reduce the overall structural density while enhancing interlayer bonding through fiber interactions, resulting in excellent interfacial toughening at the STW interfaces. It is worth noting that the performance of the hybrid lay-up system largely depends on the lay-up ratio design of carbon fiber STW and NW. Jiang et al. [19] investigated the shear resistance of anti-sandwich structured composites and proposed that the anti-sandwich structure can significantly enhance the shear strength of the overall structure. The surface structure formed by hybrid laminating STW and NW can effectively reduce microcracks induced by out-of-plane concentrated loads and demonstrate good load transfer capability. However, while existing research has focused on their shear properties, the repeated impact resistance of the anti-sandwich structure has not yet been effectively studied.

Therefore, this study investigated the resistance to repeated impacts of the anti-sandwich structure, addressing the issues that the damage patterns under repeated impact loading remained unclear and the damage mechanisms were not yet determined. Through the design of hybrid laminates composed of STW and NW, the study revealed the evolution of the anti-sandwich structure under repeated impacts.

In this study, a lightweight carbon fiber anti-sandwich laminated structure with a hybrid effect was developed, and the composites were fabricated using vacuum-assisted resin transfer molding (VARTM). The load-bearing characteristics and damage modes of the structure were investigated through repeated impact tests. The findings provide valuable insights for the design of lay-up structures and the lightweighting of composite materials in engineering applications.

## 2. Design and Preparation of Carbon Fiber STW Stitched Composites

### 2.1. Materials

In this study, STW spread by a T700-12K carbon yarn (Tianjin Anglin Maofeng High-tech Materials Co., Ltd., Tianjin, China) with an areal density of 80 g/m^2^ and NW (Jiangsu Tianniao High Technology Co., Ltd., Wuxi, China) with an areal density of 50 g/m^2^ were utilized. Additionally, T300-1K carbon fiber (Taiyuan Iron & Steel Co., Ltd., Taiyuan, China) served as the stitching yarn. The matrix system consisted of bisphenol A-type epoxy resin 6349A and amine curing agent 6349B (Shenzhen NO.1 Advanced Materials Co., Ltd., Shenzhen, China).

### 2.2. Lay-Up Design and Preform Preparation

**Figure 1:**
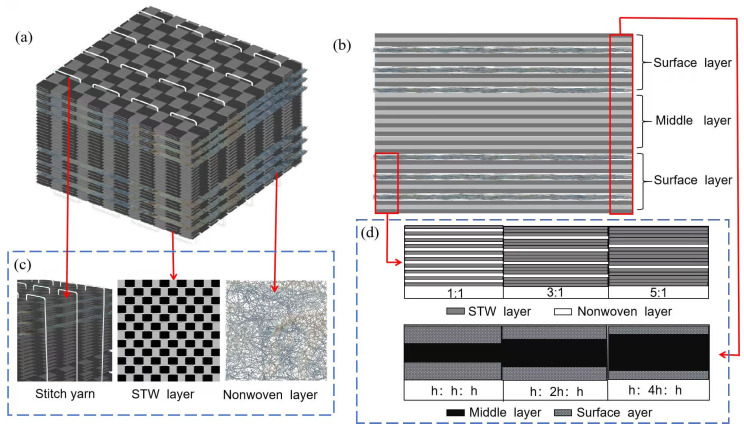
(**a**) Schematic diagram of the lay-up structure, (**b**) side view, (**c**) representation of each component and (**d**) different specifications of STW stitched composite.

The preforms were divided into two distinct regions along the thickness direction, with a core load-bearing area serving as the primary load-bearing region and lightweight surface layers on the upper and lower surfaces. The core layer consisted of continuous layers of STW and was composed of (STW_i_ + NW_j_)_n_ layers (where i is the number of spread-tow woven layers, j is the number of nonwoven layers, and n is the number of cycles of the hybrid unit). Let the single-side surface layer thickness be “h” and the core layer thickness be “mh”, where “m” is a multiplicative factor. All specimens were designated following the nomenclature “Si_mh”. To investigate the effect of structural variations on performance, five sandwich preforms with distinct configurations were fabricated. Figure 1 shows a schematic diagram of the lay-up structure. A single-ply STW preform with uniform thickness was designed for the control experiment and was named STWS.

The preform was fabricated by first stacking two-dimensional fabrics into an 8 mm thick laminated structure. Subsequently, aluminum capillary tubes with a diameter of 0.8 mm were inserted into the pore channels to guide the subsequent stitching alignment. Double strands of T300-1K carbon fiber were used as stitching threads to perform serpentine stitching in the thickness direction with a stitch density of 5 mm × 5 mm. All preform structures are shown in Table 1.

### 2.3. Fabrication of Carbon Fiber STW Stitched Composites

The carbon fiber STW stitched composites were fabricated using the VARTM process. The prepared preform was sealed in a vacuum bag. The epoxy resin and curing agent were homogeneously mixed at a mass ratio of 100:50. Subsequently, the resin mixture was infused into the preform under a vacuum pressure of −0.095 MPa. After the preform was completely infused, the composite was cured in an oven at 80 °C for 3 h. After curing, the composites were cooled to room temperature, demolded, and cut into specimens using a metallographic cutting machine for subsequent testing.

## 3. Experimental Methods

**Figure 2:**
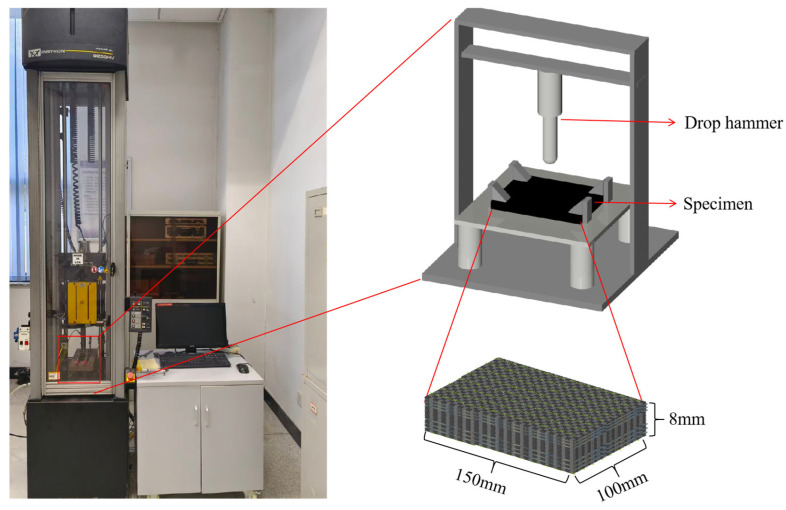
Schematic diagram of the drop-weight impact testing machine.

Repeated impact tests were performed using a 9250HV drop-weight impact testing machine (INSTRON CORPORATION, Boston, MA, USA) following the ASTM D7136 [20] standard (Figure 2). The dimensions of specimens were 150 mm × 100 mm × 8 mm, and the drop weight was 7.136 kg. The impact drop height was adjusted to achieve the preset impact energy, with two impact energies of 50 J and 100 J set to evaluate the repeated impact resistance and damage process of the composites under different energy levels (50 J and 100 J). The specimens were subjected to 20 impacts at 50 J and 3 impacts at 100 J. The tests were terminated and final data were collected when the predetermined number of impacts was reached or when perforation occurred. A profilometer was used to record the surface morphology after key impact counts, and the damage behavior and progressive damage process of the overall structure were analyzed.

## 4. Results and Discussion

### 4.1. The Load-Bearing Behavior of STW Stitched Composites Under Different Impact Energies

**Figure 3:**
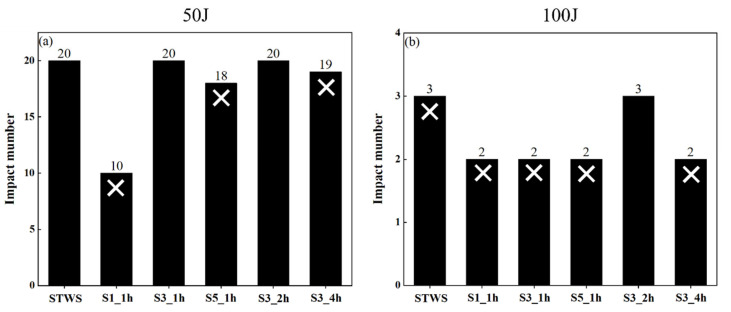
Number of repeated impacts under different impact energies: (**a**) 50 J, (**b**) 100 J.

Figure 3 presents the number of impacts sustained by each structure at different impact energies. The white X marks on the bar chart denote perforation of specimens on the final impact. Since drop-weight impact leading to specimen perforation indicates that the specimen has absorbed impact energy exceeding its load-bearing capacity and thus results in the final failure mode in the impact test, subsequent experiments were discontinued. Specifically, under 50 J impact energy, S1_1h, S5_1h, and S3_4h reached their maximum damage tolerance at the 10th, 18th, and 19th impacts, respectively, failing to withstand additional impacts and thus suffering perforation damage. Under 100 J impact energy, however, only the S3_2h specimen retained a certain load-bearing capacity after 3 impacts. This suggested that reasonable hybrid structures play a positive role in enhancing the repeated impact load-bearing capacity of the composite materials.

**Figure 4:**
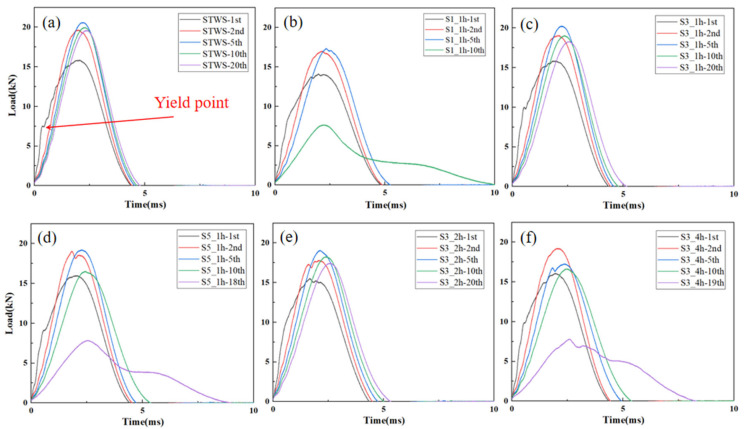
Load–time curves of different specimens under 50 J repeated impact: (**a**) STWS, (**b**) S1_1h, (**c**) S3_1h, (**d**) S5_1h, (**e**) S3_2h, (**f**) S3_4h.

Figure 4 presents the load–time curves acquired from repeated impacts at 50 J for all specimens. It can be observed that the specimens with different structures exhibited similar trends in load–time curves under repeated 50 J impacts, with the total load duration lengthening and the peak load occurring later as the number of impacts increased. This was mainly because each impact caused minor internal damage to the materials, which gradually accumulated and resulted in plastic deformation. The peak load of the first impact was lower than the maximum loads of the subsequent impacts, and a distinct yield point appeared during the loading phase. This behavior occurred because the first impact caused surface denting and matrix cracking, and the activation of cracks and defects reduced the strength and stiffness of the materials under the first impact. With the exception of S3_4h, the other five structures reached peak loads during the fifth impact, with peak loads of 20.60 kN, 17.33 kN, 20.24 kN, 19.21 kN, and 19.06 kN, respectively. At this point, crack propagation reached a critical level and stiffness of the materials peaked, suggesting stable performance of the materials immediately prior to perforation. S3_4h reached a maximum load of 19.20 kN during the second impact, exhibiting a smooth load–time curve. This was because the thickness of the hybrid lay-up in the surface layer area of S3_4h was small. After the first impact, the compression space of the surface hybrid layer was smaller, the strength of materials recovered faster, and the core layer participated in energy absorption by bearing loads earlier. This behavior could be attributed to thinner hybrid surface layers. After the first impact, the NW layers in the surface layers had limited space for further compression, allowing faster strength recovery and earlier engagement of the core layer in load-bearing and energy absorption.

**Figure 5:**
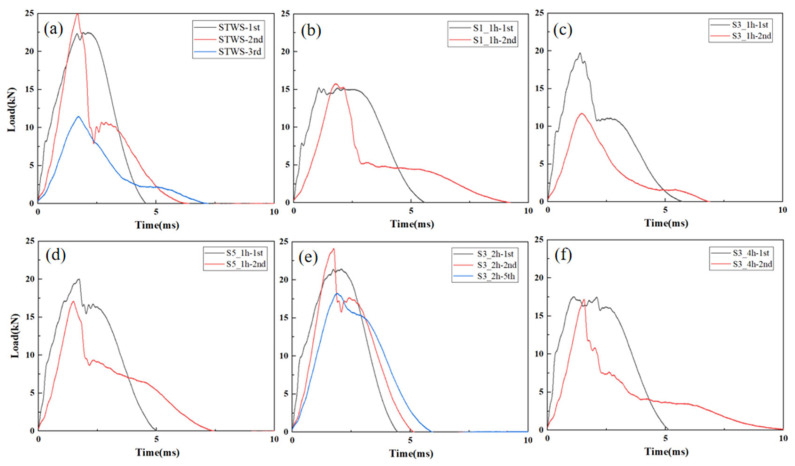
Load–time curves of different specimens under 100 J repeated impacts: (**a**) STWS, (**b**) S1_1h, (**c**) S3_1h, (**d**) S5_1h, (**e**) S3_2h, (**f**) S3_4h.

The load–time curves resulting from repeated 100 J impact tests on the specimens are illustrated in Figure 5. Similar to the behavior under 50 J impact, the load–time curves of all samples exhibited a yield point during the initial stage of the first impact. This phenomenon was attributed to matrix cracking initiated by contact with the impact head, which reduced the overall structural stiffness. STWS (Figure 5a) and S3_2h (Figure 5e) had higher load capacities, with maximum loads of 22.50 kN and 21.44 kN in the first impact, respectively, exhibiting better impact resistance. These two structures (STWS and S3_2h) had rebound time < 5 ms, withstood higher short-term loads, and showed higher energy absorption efficiency and good elastic recovery. The peak loads of S1_1h, S3_1h, S5_1h, and S3_4h were 15.24 kN, 19.76 kN, 20.03 kN, and 17.53 kN, respectively, with rebound durations all exceeding 5 ms and peak load durations being longer. This may mean that more energy was absorbed during the impact, but the lack of impact resistance led to more plastic deformation in the materials. For S3_1h, there was a significant drop in load after the peak load, indicating that delamination damage or even fiber breakage occurred inside the material. The residual stress from the first impact affected the remaining load-bearing capacity of the materials. The rebound time of all specimens in the second impact increased, indicating that stiffness of the materials decreased after the first impact, with varying degrees of plastic deformation. Specimens S1_1h, S3_1h, S5_1h, and S3_4h exhibited reduced peak loads during the second impact, with brittle fractures and perforations occurring in these materials and the failure of their core load-bearing structures (Figure 5b–d,f). The peak loads of STWS and S3_2h increased in the second impact, with maximum loads reaching 25.00 kN and 24.14 kN, respectively. The sudden drop in load of STWS after the peak indicated that fiber breakage was the dominant damage mode in this process, while the load drop of S3_2h after the peak indicated that delamination of the core load-bearing layer was the dominant damage mode. After the third impact, the peak load of STWS decreased by 54.14% with perforation damage, while S3_2h underwent damage propagation based on the originally accumulated damage, with the core load-bearing layer not completely failing, resulting in the best overall load-bearing capacity.

**Figure 6:**
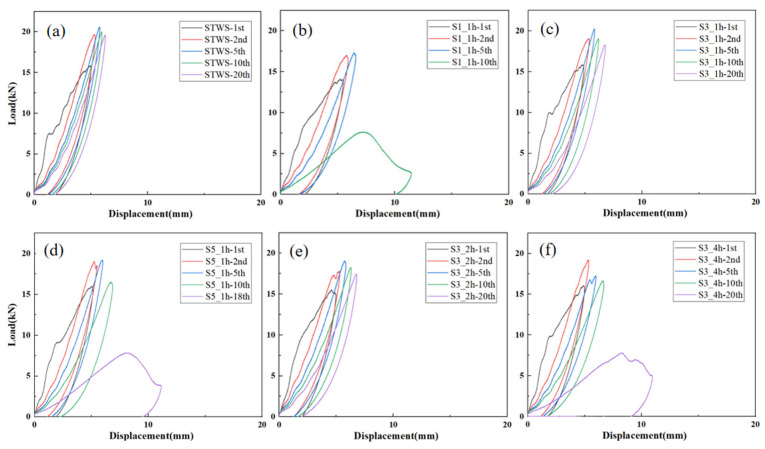
Load–displacement curves of different specimens under 50 J repeated impacts: (**a**) STWS, (**b**) S1_1h, (**c**) S3_1h, (**d**) S5_1h, (**e**) S3_2h, (**f**) S3_4h.

Figure 6 presents the load–displacement curves of the specimens under repeated 50 J impacts. As the number of impacts increased, plastic deformation accumulated, along with a gradual increase in maximum displacement and a corresponding decrease in flexural stiffness. The peak load during the initial impact was lower than the maximum residual loads observed in subsequent impacts. Prior to reaching the peak load, the force response exhibited considerable fluctuation, which was due to the generation of matrix cracks and indentations. The percentage increases in maximum displacement for impact cycles in the ranges of 1–5, 5–10, and 10–15 are presented in Table 2. The maximum displacement exhibited a relatively low increment in the 5–10 and 10–15 impact cycles. Among them, the high growth rate of S1_1h was attributed to the occurrence of perforation during the 10th impact. After 15 impacts, S5_1h and S3_4h reached impact damage tolerance due to accumulated plastic deformation, exhibiting perforations during the 18th and 19th impacts, respectively, with displacements exceeding 10 mm (Figure 6d,f).

The low displacement increments of STWS, S3_1h, and S3_2h indicated that these structures had outstanding repeated load-bearing capacity under this impact energy (Figure 6a,c,e). After 20 impacts, the residual maximum impact loads were 19.58 kN, 18.29 kN, and 17.43 kN, respectively, with maximum displacements increasing by 25.2%, 38.57%, and 37.10% compared to the 1st impact. Due to its high fiber content and more yarn interlacing, the STWS specimen exhibited the highest average stiffness of 3.29 kN/mm and provided better stress release paths during impacts. Meanwhile, S3_1h and S3_2h also maintained good stiffness, with average stiffness values of 3.06 kN/mm and 2.91 kN/mm, respectively. The 3:1 hybrid lay-up exhibited higher material toughness and stiffness under 50 J impact energy compared to the 5:1 hybrid lay-up, with less accumulated interfacial cracking under low-energy and high-frequency impacts. This was because although a higher content of long fibers in the surface layers could enhance overall stiffness, it impaired energy dissipation through structural slip, thus reducing overall impact resistance. The excessively high stiffness degradation rate of S3_4h indicated that the core layer participated in load-bearing prematurely, leading to micro-delamination damage that affected the overall stiffness of the materials.

**Figure 7:**
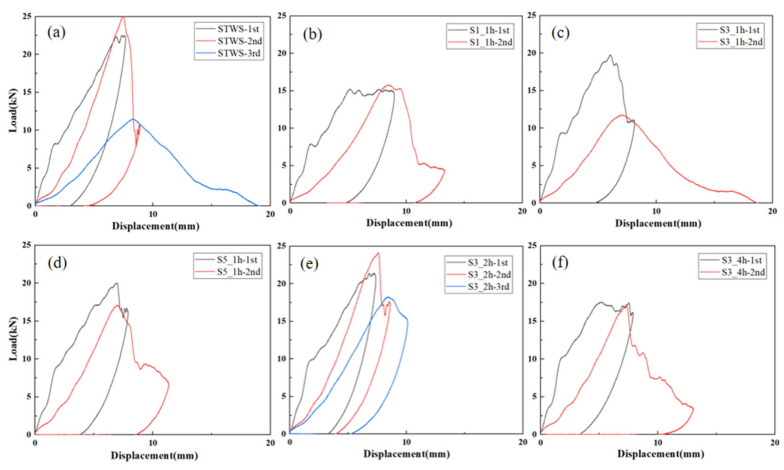
Load–displacement curves of different specimens under 100 J repeated impacts: (**a**) STWS, (**b**) S1_1h, (**c**) S3_1h, (**d**) S5_1h, (**e**) S3_2h, (**f**) S3_4h.

**Figure 8:**
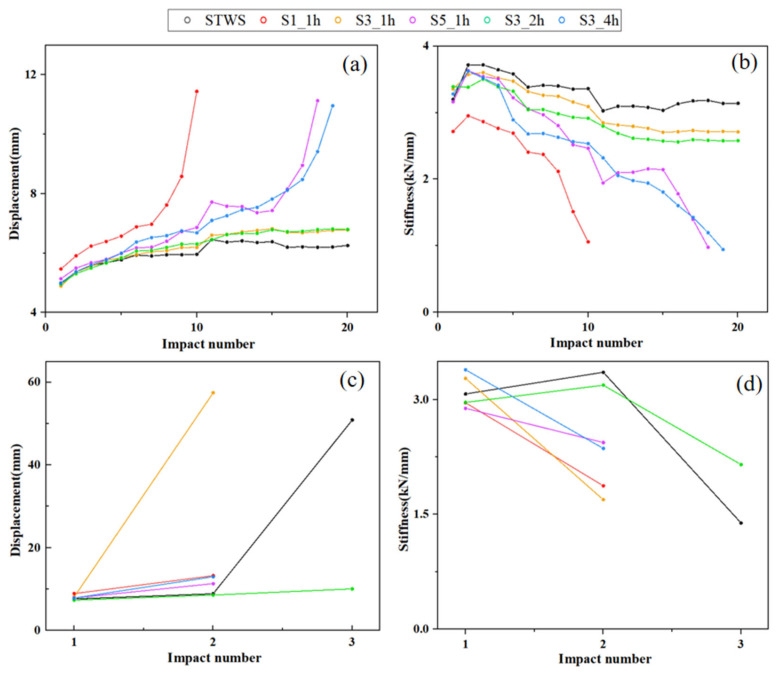
(**a**) Variation curves of maximum displacement and (**b**) variation curves of bending stiffness under 50 J impact energy; (**c**) Variation curves of maximum displacement and (**d**) variation curves of bending stiffness under 100 J impact energy.

Figure 7 presents the load–displacement curves of the specimens under repeated 100 J impacts. STWS and S3_2h demonstrated superior impact resistance, exhibiting higher peak loads and minimal internal damage during the initial impact. In contrast, the other four specimens exhibited pronounced vibrations near the maximum load, along with abrupt load drops, as shown in Figure 7c,d. Table 3 presents the relative percentage increase in maximum displacement of the samples subjected to 100 J repeated impacts. It can be observed that after the second impact, except for STWS and S3_2h (which exhibited no perforation damage), the relative growth rates of maximum displacement of all other specimens exceeded 40%. This provides numerical evidence of perforation damage. The maximum displacements of S1_1h, S3_1h, S5_1h, and S3_4h exceeded 10 mm, indicating severe perforation damage. Among them, S3_1h suffered the most serious damage, with a maximum displacement close to 20 mm, suggesting that specimens with a core layer thickness of 1h could not provide effective repeated impact protection under 100 J impact energy. S3_4h exhibited insufficient energy absorption through interfacial delamination in its toughened surface zone during the first impact. As a result, micro-delamination damage accumulated in the core load-bearing layer, leading to a reduction in the residual strength of the material. The flexural stiffness of S1_1h, S3_1h, S5_1h, and S3_4h decreased by 36.61%, 48.31%, 15.28%, and 26.22%, respectively, during the second impact. STWS and S3_2h retained residual strength after the second impact, exhibiting the smallest displacement increments (Figure 8c) and stiffness increases of 9.12% and 7.70%, respectively (Figure 8d). This indicated that the first impact failed to compromise the primary structure of the materials and resulted in limited fiber damage. Under subsequent impacts, the maximum load of STWS decreased by 54.14%, whereas that of S3_2h declined by only 24.43% during the third impact. This suggested that the S3_2h structure retained greater residual strength, confirming the significant benefit of a rational hybrid lay-up for improving impact resistance.

### 4.2. Failure Mode Analysis of STW Stitched Composites

**Figure 9:**
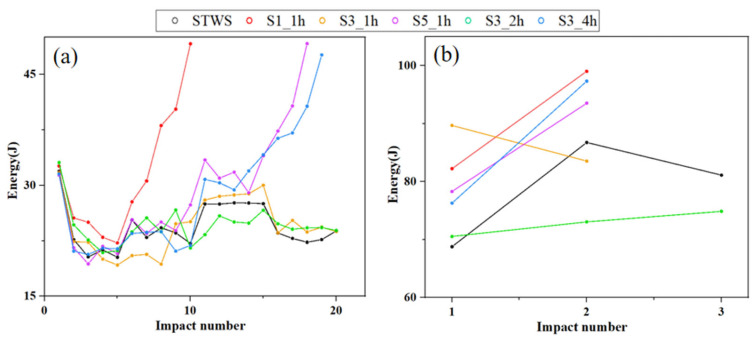
Energy absorption curves under repeated impacts with different energy: (**a**) 50 J, (**b**) 100 J.

**Figure 10:**
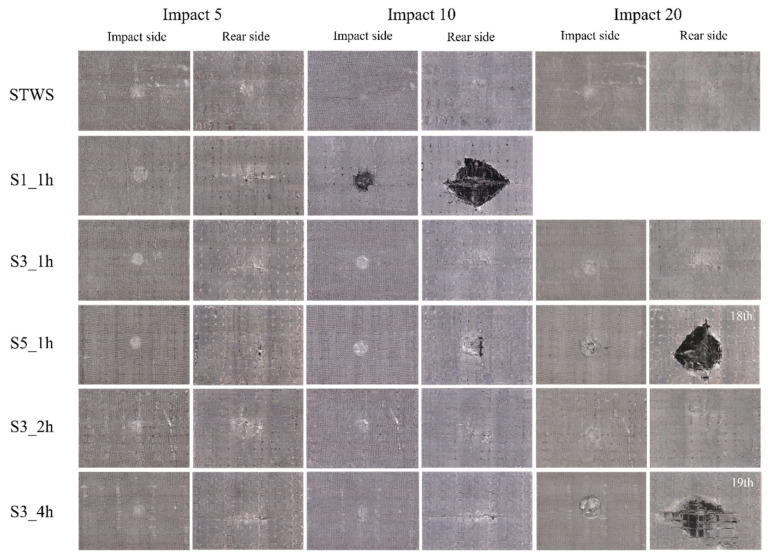
Damage morphology of the front and back sides of the composite under repeated impacts with 50 J energy.

Figure 9 presents the energy absorption statistics of the specimens under repeated impacts. As shown in Figure 9a, under a single 50 J impact, the energy absorption capacity of all structures exhibited a high degree of similarity. The composites were in a damage accumulation stage at this impact energy, absorbing energy through plastic deformation and matrix fracture rather than fiber breakage. The energy absorption of the first impact was higher than that of subsequent impacts. This was because the main damage modes of the first impact were matrix cracking and delamination between fiber layers. As the number of impacts increased, the damage modes evolved into fiber breakage and fiber pull-out, which absorbed a large amount of impact energy and eventually led to the penetration of the overall structure. The damage to the front surfaces of the materials after 20 impacts was not obvious; only matrix cracks and indentations at stress concentration points were observed on the surfaces (Figure 10). S1_1h contained the largest number of short fibers, which limited its effective load-bearing capacity. Additionally, the single layer of STW between the NW layers failed to provide sufficient structural support. During the 5th to 10th impacts, internal fibers broke sequentially, resulting in perforation during the 10th impact, with diamond-shaped protruding damage on the back surface. Therefore, a 1:1 hybrid lay-up ratio and an overly thin core layer failed to provide reliable load-bearing performance under impact loading. To a certain extent, the 3:1 hybrid lay-up exhibited higher material toughness and stiffness under 50 J impact energy. However, the core layer of S3_4h participated in load-bearing prematurely, leading to the accumulation of delamination in the core layer. Most of the energy was absorbed through delamination damage, resulting in overall stiffness degradation. Perforation occurred after the 19th impact, causing large-area bulging and unidirectional cracks. By optimizing the core layer thickness and fabric hybridization, progressive damage can be achieved, significantly improving the impact resistance of lightweight structures.

**Figure 11:**
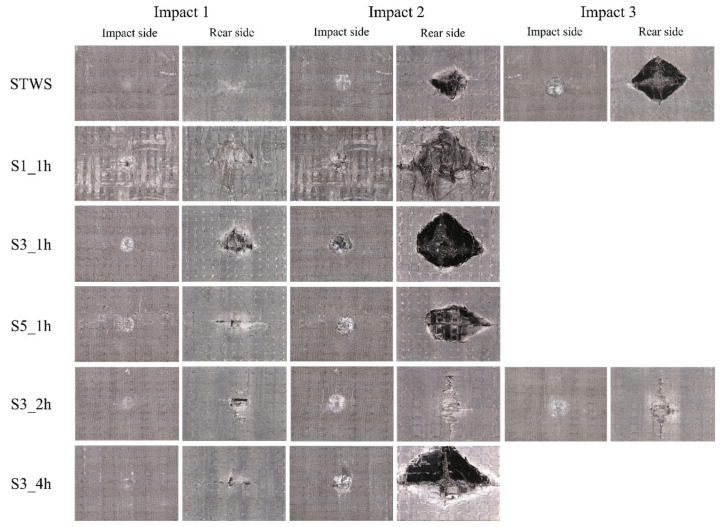
Damage morphology of the front and back sides of the composite under repeated impacts with 100 J energy.

Figure 9b shows the energy absorption changes under repeated impacts at 100 J energy, with only S3_2h remaining unperforated after three impacts. The absorbed energy increased with the number of impacts, indicating that under high impact energy conditions, the primary energy dissipation mechanisms were global structural delamination, matrix cracking, and fiber breakage on the backs of the materials (Figure 11). Statistics on the back damage areas at each impact stage are presented in Table 4. The comparison between the anti-sandwich structure proposed in this study and other existing studies is presented in Table 5. Due to the variations among different studies, the closest impact energies and optimal structures from these studies were selected for comparison. Notably, compared with the existing aluminum honeycomb sandwich structure, Nomex honeycomb sandwich structure, corrugated sandwich structure, and traditional laminated plates, the S3_2h withstands more than 20 impacts under an impact energy of 50 J and more than 3 impacts under an impact energy of 100 J, exhibiting a competitive performance in withstanding repeated impacts. Additionally, its damage mode is more controllable, mainly manifested as progressive interfacial cracking in the mixed zone and final fracture of the core layer, rather than sudden penetrating failure. This indicates that the anti-sandwich structure has significant structural advantages and damage tolerance in terms of resistance to repeated impacts, making it suitable for application scenarios with high impact risks. After the first impact, the STWS specimen exhibited the lowest energy absorption, with damage predominantly localized within the material interior. Specifically, its outer surface damage area was smaller in comparison to that of other specimens, and the measured cross-sectional area of its protruding region reached 194.23 mm^2^. This phenomenon could be attributed to the fact that the STWS specimen featured the highest number of interface layers, which facilitated the generation of more micro-delamination damage at the load-application location. Compared to STWS, the other five structural specimens exhibited higher energy absorption levels after the first impact, indicating more severe initial damage. The main reason was that the upper and lower surfaces of the materials were subjected to compressive and tensile loads, respectively, under impact. The initial fracture of the NW layers triggered interface debonding to absorb most energy, sparing the core load-bearing layer from significant damage. Owing to higher content of NW layers, the S1_1h and S3_1h specimens exhibited more severe protruding damage on back surfaces. Specifically, both specimens displayed elliptical protruding damage, with measured damage areas reaching 501.55 mm^2^ and 300.17 mm^2^, respectively. This indicated that fewer STW layers provided insufficient long fibers to disperse loads in-plane, concentrating stress waves in the out-of-plane direction and causing back-surface damage. After the second impact, all specimens with a core layer thickness of 1h suffered brittle fractures due to insufficient load-bearing capacity. The irregular fracture surface of S1_1h was mainly attributed to its highest content of short fibers, most of which were distributed near the surface. Additionally, the protruding NW layers with random fiber orientations resulted in the fracture of the surface and fiber pull-out.

The entire structure of S3_1h was penetrated by the impact hammer, with cross cracks appearing at the back protrusions, which were caused by the fracture of warp and weft yarns at stress concentration points during impact. The presence of more STW layers on the back of S5_1h created a significant load-bearing mismatch with the NW layers, resulting in more severe toughening layer delamination. The surface fibers were not completely broken, but the protrusion volume was larger. STWS absorbed 86.75 J of energy during the second impact, indicating that most energy was absorbed through the propagation of original cracks and the generation of new interfacial delamination inside the material. Excessive interfacial failure severely affected the overall stiffness of the material, eventually leading to penetration after the third impact, with a final damage area of 593.32 mm^2^. This result of damage accumulation was consistent with the structural characteristics of STW composites being prone to sudden brittle fracture. Damage to the S3_2h specimen from the first impact was localized in the toughening zone, specifically manifesting as matrix cracking on its lower surface and partial fiber breakage (Figure 11). Additionally, longitudinal cracks formed along the positions of suture holes, while protrusions were minimally obvious, and the measured damage area was only 107.56 mm^2^. In the core load-bearing zone, no visually obvious delamination was exhibited, thus retaining the specimen’s overall stiffness. The second impact caused damage propagation based on the initial damage, with obvious extensions of back cracks and more fiber breakage observed. At this point, the toughening zone provided a good energy absorption space, avoiding premature delamination damage in the core load-bearing layer. After the third impact of the S3_2h specimen, the measured damage area reached 359.52 mm^2^. The main structure of specimen underwent extensive delamination to absorb energy, though no penetration was observed. This finding indicated that a reasonable hybrid lay-up design for the composites enabled them to achieve higher damage tolerance while maintaining a certain level of material stiffness.

## 5. Conclusions and Prospects

This study developed an anti-sandwich structure composed of STW layers as the load-bearing core and hybrid STW/NW fabrics as lightweight surface layers, achieving weight reduction without compromising structural integrity. Repeated impact tests were conducted to evaluate its damage tolerance. The main conclusions are as follows:

(1) For the anti-sandwich specimens, there exists an optimal balance in the hybrid ratio between the core and surface layers. Specifically, the S3_1h configuration (core-to-surface thickness ratio = 2:1, STW/NW hybrid ratio = 3:1) exhibited the best overall performance in repeated impact resistance.
(2) Increasing the core layer proportion enhances overall stiffness of the structure but reduces damage tolerance.
(3) The lightweight surface layers not only reduce the overall density of the composite structure but also facilitate damage dispersion, as evidenced by the progressive interfacial cracking and energy absorption mechanisms observed under impact loading.
(4) Structural damage initiates as interfacial cracking in hybrid zones, which is then followed by core layer failure. This phased damage evolution positively contributes to preserving the residual strength of the materials.

Compared to conventional metals [23] or laminates [24], this structure maintains impact resistance while reducing overall density, demonstrating significant potential for applications in high-load and high-impact scenarios such as aircraft wings [25], engine cold-section blades [26], and rapid-deployment structures [27].

## Figures and Tables

**Table 1:** Parameters of the samples.

Sample	Surface Lay-Up Parameters	Core Layer Lay-Up Parameters	Fiber Volume Fraction (%)	Average Thickness (mm)
STWS	-	STW_90_	46.8	7.05
S1_1h	(STW_1_/NW_1_)_10_	STW_29_	32.4	7.99
S3_1h	(STW_3_/NW_1_)_6_	STW_29_	37.3	7.84
S3_2h	(STW_3_/NW_1_)_5_	STW_45_	40.5	7.94
S3_4h	(STW_3_/NW_1_)_3_	STW_58_	41.8	7.13
S5_1h	(STW_5_/NW_1_)_4_	STW_29_	38.7	7.35

**Table 2:** Relative increase rate of maximum displacement between impact numbers under 50 J impact energy.

Sample	Relative Increase in Maximum Displacement (%)		
	5th/1st	10th/5th	15th/10th
STWS	15.60	3.11	7.21
S1_1h	20.29	73.86	-
S3_1h	19.39	5.81	10.18
S5_1h	16.73	14.5	8.15
S3_2h	17.54	3.77	7.61
S3_4h	20.00	11.69	16.89

**Table 3:** Relative increase rate of maximum displacement between impact numbers under 100 J impact energy.

Sample	Relative Increase in Maximum Displacement (%)	
	2nd/1st	3rd/2nd
STWS	13.83	119.14
S1_1h	46.72	-
S3_1h	129.50	-
S5_1h	43.75	-
S3_2h	13.87	19.90
S3_4h	65.17	-

**Table 4:** Statistics on back damage areas at each stage of impact.

Sample	Back Damage Area Under 100 J Impact Energy (mm^2^)			Back Damage Area Under 50 J Impact Energy (mm^2^)		
	1st	2nd	3rd	5th	10th	20th
STWS	194.23	387.14	593.31	97.29	185.92	282.07
S1_1h	501.54	604.29	-	182.83	677.90	-
S3_1h	300.17	587.50	-	255.50	283.88	355.38
S5_1h	481.00	680.21	-	143.94	345.69	612.62
S3_2h	107.55	408.58	767.43	209.14	251.12	359.52
S3_4h	283.06	753.16	-	149.15	455.58	1013.11

**Table 5:** Resistance capacity of composite materials with different structures against repeated impacts.

Structure	Impact Energy	Number of Impacts	Damage Modes
This work (S3_2h)	50 J	>20	Interfacial cracking at hybrid zones, followed by core-layer failure
	100 J	>3	
Sandwich structure with aluminum honeycomb core [21]	40 J	4	Skin penetration, fiber breakage, and debonding between core and skin
	70 J	2	
Sandwich structure with Nomex honeycomb core [17]	6 J	4	The impact fully penetrated through both skins
	8 J	1	
Corrugated sandwich structure [22]	15 J	5	Two penetrations within a short time interval
	50 J	1	
Laminates [9]	15 J	12	Progressive failure involving interlaminar delamination, fiber fracture, and penetration

## Data Availability

The data presented in this study are available on request from the corresponding author due to privacy.
